# Immediate Weight-Bearing after Ankle Fracture Fixation

**DOI:** 10.1155/2015/491976

**Published:** 2015-02-16

**Authors:** Reza Firoozabadi, Emily Harnden, James C. Krieg

**Affiliations:** ^1^Department of Orthopaedics and Sports Medicine, Harborview Medical Center, University of Washington, 325 9th Avenue, P.O. Box 3595798, Seattle, WA 98104, USA; ^2^Rothman Institute, Philadelphia, PA 19107, USA

## Abstract

We believe that a certain subset of surgical ankle fracture patients can be made weight-bearing as tolerated immediately following surgery. Immediate weight-bearing as tolerated (IWBAT) allows patients to return to ambulation and activities of daily living faster and may facilitate rehabilitation. A prospectively gathered orthopaedic trauma database at a Level 1 trauma center was reviewed retrospectively to identify patients who had ORIF after unstable ankle injuries treated by the senior author. Patients were excluded if they were not IWBAT based on specific criteria or if they did meet followup requirement. Only 1/26 patients was noted to have loss of fixation. This was found at the 6-week followup and was attributed to a missed syndesmotic injury. At 2-week followup, 2 patients had peri-incisional erythema that resolved with a short course of oral antibiotics. At 6-week followup, 20 patients were wearing normal shoes and 6 patients continued to wear the CAM Boot for comfort. To conclude, IWBAT in a certain subset of patients with stable osteosynthesis following an ankle fracture could potentially be a safe alternative to a period of protected weight-bearing.

## 1. Introduction

Ankle fractures are among the most common injuries treated by orthopaedic surgeons [1, 2]. Open anatomic reduction and internal fixation are routinely advocated for displaced, unstable ankle fractures [3–5]. Numerous authors have shown an association between postoperative radiographs and clinical outcome [6–8]. Recently, emphasis has been placed on functional outcome and recovery. Faster return of function and return to work are related to rehabilitation strategy. Following operative treatment of ankle fractures, most physicians advocate a period of nonweight-bearing followed by partial progressive weight-bearing.

The primary goals of fracture surgery and postoperative regimen are to minimize disability from injury. A secondary goal is to minimize the period of convalescence and thus maximize function as expediently as possible, given the usual considerations to risk and benefit. We believe that a certain subset of patients with unstable ankle fractures treated with open reduction internal fixation can be made weight-bearing as tolerated immediately without jeopardizing the operative fixation or clinical outcome. We assume that earlier weight-bearing will allow patients to return to their activities of daily living quicker, with an overall easier time during convalescence.

## 2. Materials and Methods

**Figure 1:**
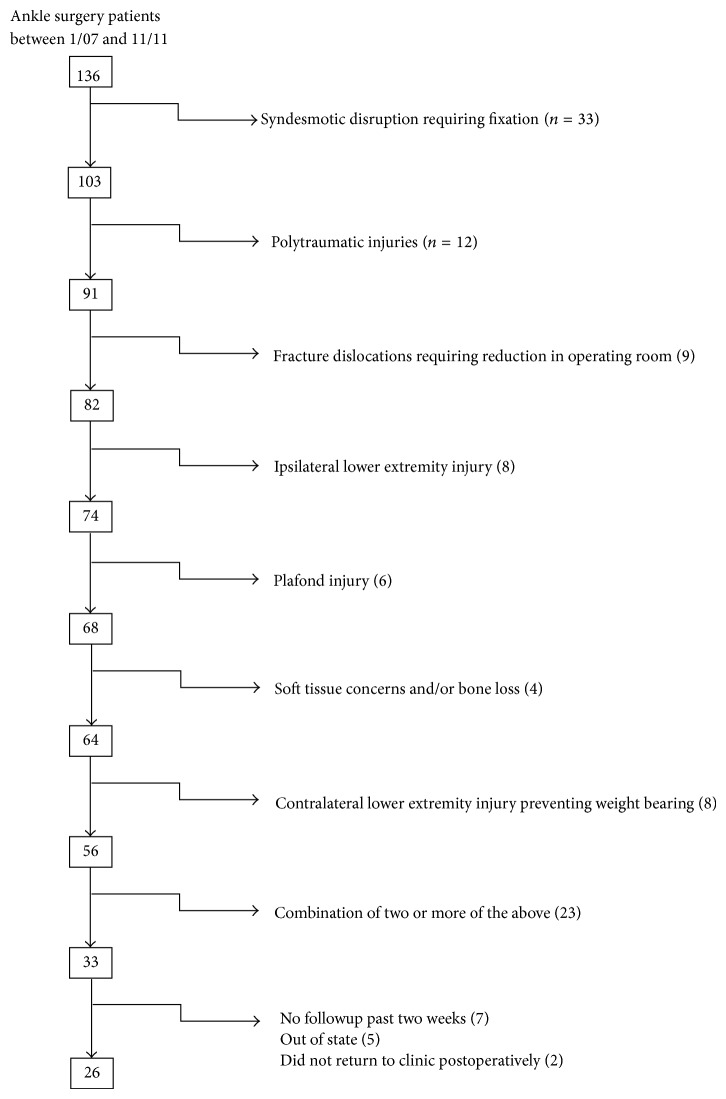
Exclusion diagram for 136 patients with ankle fractures over 23-month period.

This study was approved by our institutional review board. A prospectively gathered orthopaedic trauma database at a Level 1 trauma center was reviewed retrospectively to identify patients who had sustained unstable ankle injuries treated by the senior author between January 2007 and December 2011. A total of 136 skeletally mature patients underwent ankle surgery, 33 of which were allowed immediate weight-bearing as tolerated (IWBAT) in the acute postoperative period. Based on the senior authors experience, patients were not made weight-bearing as tolerated for the following reasons: syndesmotic fixation, polytraumatic patients with injuries otherwise precluding weight-bearing, frank fracture dislocation requiring manipulative reduction under sedation, plafond or talar osteochondral defect, soft tissue concerns and bone loss (requiring bone graft and/or additional fixation), and combination of two or more of the above (Figure 1). Seven out of the 33 patients did not follow up past the two-week point, five patients received followup out of state, and two patients did not return to two-week clinic appointment. As a result, 26 patients were included for assessment in this study.

Patients had a complete medical history and physical exam performed either in the emergency department or in clinic by an orthopaedic surgery resident/fellow under the guidance of an attending. Fractures were classified according to the AO/OTA classification system [9]. Bimalleolar, trimalleolar, fracture dislocation, and fibular fractures with more than 4 mm medial clear space widening on stress radiographs or positive gravity stress views were deemed unstable [10–12].

Operative protocol included open anatomic reduction and internal fixation of the fibula by resident/fellow supervised by the trauma fellowship trained the senior author. In cases in which the medial malleolus was fractured, screws or small fragment plates were used for fixation. Posterior malleolus fractures were fixed on a case-by-case basis. Although no clear indications exist for fixation of small posterior malleolus fractures, many of the small fractures and all of the larger fractures were treated operatively. The syndesmosis was reduced and held in place with two 3.5 mm screws if stress testing displayed widening after the malleoli were fixed.

Postoperative protocols were similar to all patients. Patients who were allowed IWBAT were protected in a Controlled Ankle Motion (CAM) Walker Boot. The boot was kept on at all times for the first two weeks. Patients were instructed to keep the wound dry until seen at the two-week clinic followup. At two weeks, the dressings were removed and the wound assessed. The sutures were removed and replaced with Steri-Strips. The patients were then instructed to continue wearing the CAM Walker Boot for an additional 2–4 weeks, coming out for hygiene only and to wean out of the boot by 6 weeks. Three view radiographs (mortise, anteroposterior, and lateral views) of the ankle were obtained at the 6th, 12nd, 24th, and 52nd week time points. At 6-week postoperation, the boot was discontinued if the patient had not already converted over to a shoe. Patients were offered a removable ankle stirrup to aid in weaning.

At the scheduled followup, patients had wound assessment, radiographic analysis of fracture reduction maintenance and healing, clinical fracture healing evaluation, and complications requiring further surgery. Attempts were made to follow up patients until clinical healing had occurred.

## 3. Results

Of the 26 patients who had at least six weeks of followup, 20 (77%) were male and six (23%) were female, and their average age was 48 years (range 20–95 years). The mechanism of injury included 17 low-energy falls, three motor vehicle accidents, two pedestrians struck by motor vehicles, two twisting injuries while playing sports, one fall off bicycle, and one assault. According to AO/OTA fracture classification, there were four of type-44A (4%), 21 of type-44B (81%), and one of type-44C1 which did not require syndesmotic reduction and fixation (4%). Fifteen patients (58%) were cigarette smokers, and two patients (8%) had noninsulin dependent diabetes with no peripheral neuropathy.

Lateral malleolus fixation included 20 1/3rd tubular plates (77%), four precontoured posterolateral plates (15%), and one intramedullary nail (4%). Medial malleolus fixation was required in eleven patients (42%); screws were used in ten cases (91%) and a plate in 1 case (9%). Posterior malleolus required fixation in five cases (19%). Twenty-five patients had intraoperative postfixation radiographs that displayed symmetric joint space around the talus. One patient had 1.7 mm increased lateral joint space compared to medial and superior clear space.

Average followup time was 140 days (range 40–478 days). Clinical evaluation at two weeks was noted for two patients having peri-incisional erythema that resolved with a short course of oral antibiotics (8%). At six weeks, no wound issues were noted. Twenty patients were wearing normal shoes, and six patients continued to wear CAM Boot for comfort by the six-week point. At the last clinic visit, three patients had persistent ankle stiffness, one patient had symptoms consistent with peroneal subluxation, which resolved with physical therapy, and one patient required removal of medial malleolar fixation secondary to symptomatic hardware.

**Figure 2:**
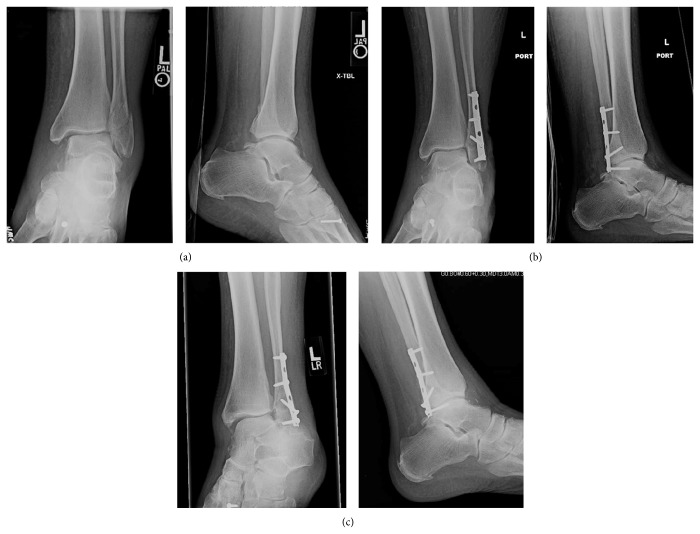
Single case of loss of reduction, suspect secondary to missed syndesmotic injury. (a) Preoperative mortise and lateral radiographs. (b) Immediate postoperative mortise and lateral radiographs. (c) 6 weeks of followup mortise and lateral radiographs.

**Figure 3:**
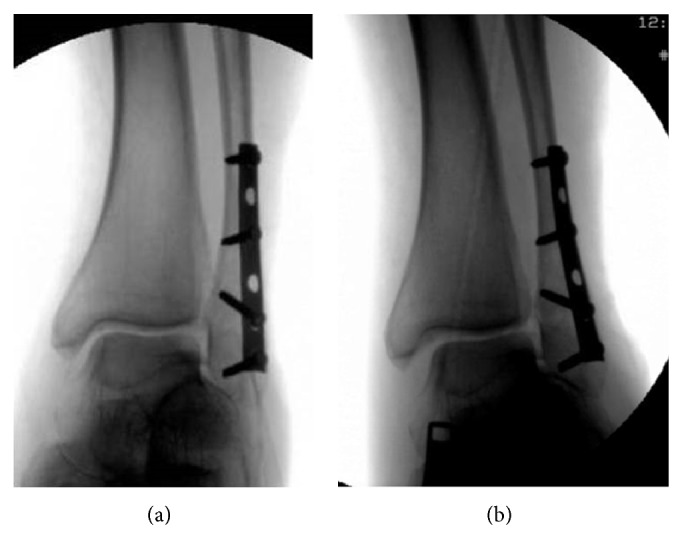
Intraoperative fluoroscopic images of failure case. (a) Preexternal rotation stress mortise view. (b) External rotation stress mortise view. Medial clear space widening suggestive of missed syndesmotic injury.

Radiographic evaluation at six weeks displayed no loss of reduction in 25 patients (96%) and one loss of reduction (4%). This was the same patient that was noted to have 1.7 mm of increased lateral joint space compared to medial and superior clear space. At the six-week interval, the lateral joint space was 4.8 greater than the medial and superior clear space (Figure 2). Intraoperative fluoroscopy images were reviewed, and it was noted that the patient had a missed syndesmotic injury (Figure 3).

## 4. Discussion

This study demonstrates that IWBAT in a certain subset of patients with stable osteosynthesis following an ankle fracture is a safe alternative to a period of protected weight-bearing. Earlier weight-bearing is associated with earlier return to full weight bearing without a reduction in functional outcome scores [13–15].

Early weight-bearing is routinely used to treat stable ankle fractures. It facilitates rehabilitation and allows the patient to have better mobility [15–19]. Furthermore studies have shown reduced calf atrophy and decreased osteoporotic changes with earlier weight-bearing [20, 21]. Simanski et al.'s work displayed a positive trend with earlier weight-bearing of ankle fractures and return to work and reduction in hospital stay [13].

This study was designed to analyze whether immediate weight-bearing after stabilization of unstable ankle fractures would result in early loss of fixation. To our knowledge, only one other group has published a series on immediate weight-bearing as tolerated after ankle fixation without a cast. Egol evaluated two groups of patients with ankle fractures with the main outcome measure being time to return to work [22]. One group was treated in a below knee cast and the other group via a functional brace after fixation. Both groups were nonweight-bearing on the affected side. Mean time from surgery to return to work was substantially shorter in the functional brace early movement group (7.6 versus 15.2 weeks). Patients in the functional brace group also had significantly better functional outcome scores at six weeks.

Ahl et al. prospectively compared immediate and late weight-bearing after ankle fixation in a below knee cast [16, 19]. Radiographic and clinical analysis at three and six months did not display a difference between the two groups. Time to return to work was not assessed. More recently, Starkweather et al. retrospectively reviewed 126 patients who bore weight in a short leg cast within 15 days after surgical repair of acute unilateral closed ankle fractures. Ninety-nine percent of the radiographs showed no loss of reduction on final followup examination [23]. Simanski et al. performed a prospective study comparing functional early weight bearing (3 weeks) to 6 weeks without weight-bearing in a below knee cast [13]. The early weight-bearing group was allowed partial weight-bearing (10–15 kg) in an Aircast Air-Stirrup Brace immediately after surgery. The patients were then allowed full weight bearing at 3 weeks if no problems were identified. Early weight-bearing patients were able to obtain full weight-bearing in advance of the delayed group (7.7 versus 13.5 weeks, *P* = 0.01). No disadvantage was noted in regard to the early weight-bearing group both clinically and radiographically.

Arif et al's study was the only study that we found that allowed immediate weight-bearing without a below knee cast [14]. This was a retrospective study with one group of patients that were allowed weight-bearing as tolerated postoperatively without a cast, and the other group of patients were placed in a cast and made nonweight-bearing for 6 weeks. Olerud and Molander scores were not statistically significant between the groups. Return to work was 55 days for the early weight-bearing group versus 91 days for the delayed weight-bearing group, which was statistically significant.

The above stated studies all suggest that earlier weight-bearing and motion would allow patients earlier return to function without any compelling disadvantage. Our findings show that patients can fully weight-bear as tolerated during the immediate postoperative period similar to patients with stable ankle fractures. Our patient group had one case of loss of reduction and fixation failure. This occurred as a result of a missed syndesmotic injury. This reaffirms the importance of identifying syndesmotic disruptions.

This study has a number of limitations inherent in any retrospective case series. The major limitation being that only a subset of patients with unstable ankle fractures was allowed immediate full weight-bearing. This discretion was set by the senior authors practice guideline, which does not allow IWBAT in polytrauma patients, cases of syndesmotic disruption, and concerns for soft tissue compromise. Additionally, while we did not exclude diabetic patients (no insulin dependent diabetic patients met inclusion criteria), one should consider not allowing patients with poorly controlled diabetes and/or peripheral neuropathy to bear weight immediately due to soft tissue healing concerns. Patients with poor bone quality and comminution should potentially also be excluded. While we did not exclude patients for these two factors they can theoretically result in early failures in patients that are allowed to bear weight immediately. Another limitation of this study is that we did not have a control group and seven patients did not have appropriate followup and therefore excluded. If all seven of these patients had loss of reduction then the failure rate would be unacceptable at 24%. Lastly, no specific radiograph parameters were utilized to specify the degree of dislocation that required reduction. Although this study does support immediate weight-bearing postoperatively for a certain subset of patients with ankle fractures, we feel that a controlled, prospective trial is warranted to look further at the influence of delayed versus immediate weight-bearing after ankle fixation surgery.

## 5. Conclusion

IWBAT in a certain subset of patients with stable osteosynthesis following an ankle fracture is a safe alternative to a period of protected weight-bearing. Earlier weight-bearing has been associated with better mobility, shorter hospital stay, and earlier return to work. Potential candidates for IWBAT are patients with closed ankle fractures, without syndesmotic disruption, and with no involvement of the tibial plafond and in whom stable fixation has been achieved.
